# Physical, Psychophysical and Psychological Predictors of Persistent or Recurrent Non‐Specific Low Back Pain: A Systematic Review and Meta‐Analysis

**DOI:** 10.1002/ejp.70358

**Published:** 2026-09-04

**Authors:** Kanya Wongwitwichote, Cho Wai Geoffrey Yu, Valter Devecchi, Michael Mansfield, Deborah Falla

**Affiliations:** ^1^ Centre of Precision Rehabilitation for Spinal Pain, School of Sport, Exercise and Rehabilitation Sciences University of Birmingham Birmingham UK

**Keywords:** chronic pain, low back pain, prediction, recurrence

## Abstract

**Background and Objective:**

Non‐specific low back pain (LBP) frequently becomes persistent or recurrent. This systematic review and meta‐analysis determined whether physical, psychophysical or psychological variables can predict persistent or recurrent non‐specific LBP.

**Database and Data Treatment:**

Studies investigating physical, psychophysical or psychological predictors of persistent/recurrent LBP, pain intensity or disability were included. MEDLINE, EMBASE, APA PsycINFO, PubMed, CINAHL Plus, Web of Science, Scopus, ZETOC and OpenGrey were searched until January 2025. Risk of bias of individual studies was assessed using QUIPS and the certainty of evidence using GRADE.

**Results:**

Fifteen studies were included, all addressing persistent LBP, examining one physical predictor domain (trunk kinematics), one psychophysical predictor domain (pain sensitivity) and seven psychological domains (depression‐distress, anxiety, fear avoidance, catastrophising, somatisation, pain coping strategy, pain self‐efficacy). Depression and distress significantly predicted persistent LBP and disability (*n* = 15,778; *β* = 0.28, 95% CI: 0.04 to 0.53, *p* = 0.024), and fear avoidance also significantly predicted persistent LBP and disability (*n* = 1105; *β* = 0.24, 95% CI: 0.00 to 0.49, *p* = 0.049), albeit both with low certainty of evidence. Enhanced temporal summation of pain was not a significant predictor of pain outcomes (*n* = 192; *β* = 0.10, 95% CI: −0.37 to 0.57), with very low certainty of evidence. Physical predictors showed limited and inconclusive evidence. Overall, the certainty of evidence ranged from very low to low across all predictor domains.

**Conclusion:**

Psychological factors, particularly depression‐distress and fear avoidance, may predict LBP persistence, although higher quality studies are needed.

**Significance Statement:**

Depression‐distress and fear avoidance are significant predictors of persistent non‐specific low back pain, while evidence for physical predictors remains limited. These findings emphasize the need for early identification of modifiable psychological risk factors.

**Trial Registration:**

PROSPERO (Registration number CRD42024599514)

**Abbreviations**
4DSQ: Four‐Dimensional Symptom Questionnaire
CPG: Chronic Pain Grade
CSQ: Coping Strategies Questionnaire
DRAM: Distress and Risk Assessment Method
FABQ‐PA: Fear‐Avoidance Beliefs Questionnaire—Physical Activity
GCPS: Graded Chronic Pain Scale
GHQ: General Health Questionnaire
HAD: Hospital Anxiety and Depression Scale
LBP: Low Back Pain
PCS: Pain Catastrophizing Scale
PHQ‐4: Patient Health Questionnaire‐4
RMDQ: Roland‐Morris Disability Questionnaire
SCL‐CD: Symptom Checklist‐core depression scale
TSK: Tampa Scale for Kinesiophobia
VAS: Visual Analogue Scale
VPMI: Vanderbilt Pain Management Inventory
Zung SDS: Zung Self‐Rating Depression Scale

## 1 Introduction

Low back pain (LBP) is a major global health problem affecting people across all ages (Chen et al. 2022; Collaborators 2024). Its incidence is projected to rise, with more than 800 million cases expected worldwide by 2050 (Collaborators 2023). LBP imposes a significant burden on individuals and healthcare systems (Agnus Tom et al. 2022; Chang et al. 2024; Fatoye, Gebrye, Mbada, and Useh 2023); as an example, in high‐income counties including European nations, annual per‐patient direct costs of LBP have been estimated at approximately USD 9,231, with total costs reaching USD 10,143 per patient (Fatoye, Gebrye, Ryan, et al. 2023). Recurrence is common and about 67% of individuals experience another episode within a year (da Silva et al. 2019) and more than half of those with chronic LBP report sudden flare‐ups (Suri et al. 2012), greatly impacting their quality of life (Tan et al. 2019). In most cases, LBP is non‐specific, meaning that no identifiable underlying spinal pathology can be established (Balagué et al. 2012). LBP may follow a recurrent course, defined as repeated episodes separated by a pain‐free interval of at least one month (Stanton et al. 2009, 2011), or a persistent course, defined as pain continuously present for 12 weeks or more (Menezes Costa et al. 2012; Wallwork et al. 2024). Identifying individuals at risk of recurrent or persistent LBP is therefore essential for targeted prevention, personalized treatment, and efficient healthcare resource allocation.

The persistence and recurrence of non‐specific LBP, the most prevalent form of LBP, are thought to be influenced by a combination of physical, psychophysical, and psychological factors. Physical impairments, including altered trunk movement patterns, poor motor control, and increased trunk muscle activation, are frequently observed in individuals with current LBP (Alkhathami and Alqahtani 2024; Alsubaie et al. 2023; Dambadarjaa et al. 2024; Ghamkhar and Kahlaee 2019; Liew et al. 2020; Moissenet et al. 2021; Šarabon et al. 2021; Krueger et al. 2025). Some of these features have been proposed as potential biomarkers capable of distinguishing individuals with LBP from asymptomatic populations (Liew et al. 2020; Moissenet et al. 2021). Moreover, such physical alterations may provide mechanistic insight into the processes underlying symptom recurrence or persistence. For example, a systematic review revealed that persistent changes in trunk muscle activity have been reported even during periods of remission in individuals with recurrent LBP (Devecchi et al. 2021), suggesting a potential role in the re‐emergence of symptoms. Similarly, deficits in endogenous pain inhibition (i.e., psychophysical changes) have been shown to endure after pain subsides, potentially predisposing individuals to recurrent episodes (McPhee et al. 2020). Nevertheless, despite growing evidence characterizing these physical and psychophysical changes in the presence of LBP, it remains uncertain whether such features can predict future LBP persistence or recurrence.

Alongside physical impairments, psychological factors play a critical role in the course of non‐specific LBP. Psychological constructs such as depression, anxiety, fear avoidance, pain catastrophizing, and low self‐efficacy have been identified as risk factors for the development and maintenance of LBP and are known to influence pain intensity, disability and treatment outcomes (Alhowimel et al. 2021; Nieminen et al. 2021; Li et al. 2025). These factors may influence LBP recurrence through several interrelated mechanisms. Where individuals with LBP also exhibit catastrophizing thoughts, the anticipation of pain may generate fear of movement (Wertli, Burgstaller, et al. 2014). Such fear‐avoidance behaviours can persist after pain resolution, gradually reducing physical fitness and leaving individuals more susceptible to recurrent LBP (Vlaeyen and Linton 2000). The presence of anxiety may further elevate the risk of LBP recurrence, as it can cause individuals to concentrate on negative perceptions of their illness, thereby reducing their capacity for adaptive coping and active participation in recovery (Jiang et al. 2022). Psychological distress may therefore not only exacerbate pain perception and but also limit engagement in the movement behaviours, thereby fostering chronicity. Nevertheless, while these factors are well‐established predictors of poor recovery and functional limitation, the extent to which they can independently predict LBP persistence or recurrence over time remains unclear. Unlike fixed demographic or structural factors, psychological factors are potentially modifiable, making their early identification clinically important for preventing persistent or recurrent LBP. Identifying such modifiable predictors is therefore of considerable clinical relevance, as it may inform targeted screening and early intervention strategies. A more integrated understanding of how physical and psychophysical domains influence recurrent or persistent non‐specific LBP, would facilitate the development of predictive models and tailoring of preventive interventions for individuals at risk. Thus, this systematic review aimed to synthesize and evaluate the certainty of evidence on whether physical, psychophysical or psychological features can predict the persistence and recurrence of non‐specific LBP.

## 2 Methods

### 2.1 Protocol Registration and Study Design

The protocol of the systematic review and meta‐analysis was registered with the International Prospective Register of Systematic Reviews (PROSPERO) under the identifier CRD42024599514 and was published (Wongwitwichote et al. 2025). Minor modifications were made to the population criteria during the review process. Persistent LBP was defined as individuals experiencing current LBP at the time of recruitment (acute, subacute or chronic) who continued to report pain at follow up assessment (i.e., persistence of pain). The report of this systematic review and meta‐analysis follows the reporting standards established by the Preferred Reporting Items for Systematic reviews and Meta‐Analyses (PRISMA) 2020 statement (Page et al. 2021).

### 2.2 Eligibility Criteria

The eligibility criteria for this systematic review were structured using the PICOS framework (Riley et al. 2019), where P represents the target population, I denotes the index predictive factor of interest, C encompasses comparator predictive factors (alternative predictive variables for comparison), O specifies the outcomes of interest and S defines the study design.

#### 2.2.1 Population

The study population included individuals aged 18 years or older with a history of non‐specific LBP. At the time of recruitment into study, two populations were considered: (1) For examining predictive factors of recurrence, studies should have included individuals in a period of remission from LBP at study entry, defined as currently symptom‐free following a prior recurrent episode of LBP. Their history of recurrent pain should have been characterized by the experience of new episodes of LBP lasting at least 24 h and reaching a minimum pain intensity equivalent to the minimal important change for the chosen scale, occurring after a pain‐free period of at least 1 month, with no maximum duration specified (Stanton et al. 2009, 2011); (2) For examining predictive factors of pain persistence, studies should have included individuals experiencing current LBP at the time of recruitment, whether it be acute, subacute or chronic.

#### 2.2.2 Index Predictive Factors

The index predictive factors in this study included physical, psychophysical and psychological predictors of non‐specific LBP persistence and recurrence. Physical predictors included any physical measure such as, but not limited, to lumbar kinematics (e.g., range of motion, speed and smoothness of movement), trunk muscle strength, trunk muscle activity. Psychophysical predictors included findings from quantitative sensory testing (QST), which involves the application of the standardized physical stimuli to evaluate somatosensory function. Psychological features included distress, anxiety, depression, fear avoidance, catastrophizing, somatization, pain coping strategies and pain self‐efficacy.

#### 2.2.3 Outcomes

The primary outcomes were recurrence of LBP for those that were recruited during remission or persistent LBP for those who were recruited with current LBP. LBP recurrence was measured as either the number of pain episodes or total days with pain during the follow‐up period, while persistent LBP was defined as the presence of pain or disability evaluated at different time points after the assessment of potential predictors, with a minimum follow‐up of 12 weeks, whereby pain reported at or beyond this point was considered to represent persistent LBP. Additionally, we included LBP intensity measured using the Visual Analog Scale (VAS), Numeric Pain Rating Scale (NPRS) or Chronic Pain Grade (CPG); low back pain incidence rates; and disability levels assessed with the Oswestry Disability Index (ODI) or Roland Morris Disability Questionnaire (RMDQ).

#### 2.2.4 Study Design

Prospective cohort and longitudinal studies involving participants with a history of non‐specific LBP were included. Eligible studies needed to document symptom recurrence or exacerbation during follow‐up and report outcomes including new pain episodes, LBP exacerbations, pain intensity or disability levels. Studies were required to examine physical, psychophysical or psychological predictors. Exclusion criteria included studies which included individuals with radicular pain, serious spinal pathologies (e.g., tumours, infections, cauda equina syndrome), rheumatological disease such as rheumatoid arthritis, or those who were pregnant. Case reports, case–control studies, publications limited to abstracts and articles not published in English were also excluded.

### 2.3 Search Strategy

An extensive literature search strategy was developed in collaboration with a University of Birmingham librarian. Multiple databases were systematically searched to identify relevant publications from inception to January 2025, including MEDLINE, EMBASE, APA PsycINFO, PubMed, CINAHL Plus, Web of Science, Scopus, ZETOC and grey literature search from Open Grey. All retrieved studies were imported into EndNote 20.6 (Clarivate Analytics, New York City, NY) before being transferred to the Rayyan web‐based screening platform (http://rayyan.qcri.org). Duplicate records were eliminated through the automated deduplication feature in EndNote, followed by additional duplicate removal within the Rayyan platform. Detailed search strategies are available in Table S1.

### 2.4 Study Selection

**FIGURE 1:**
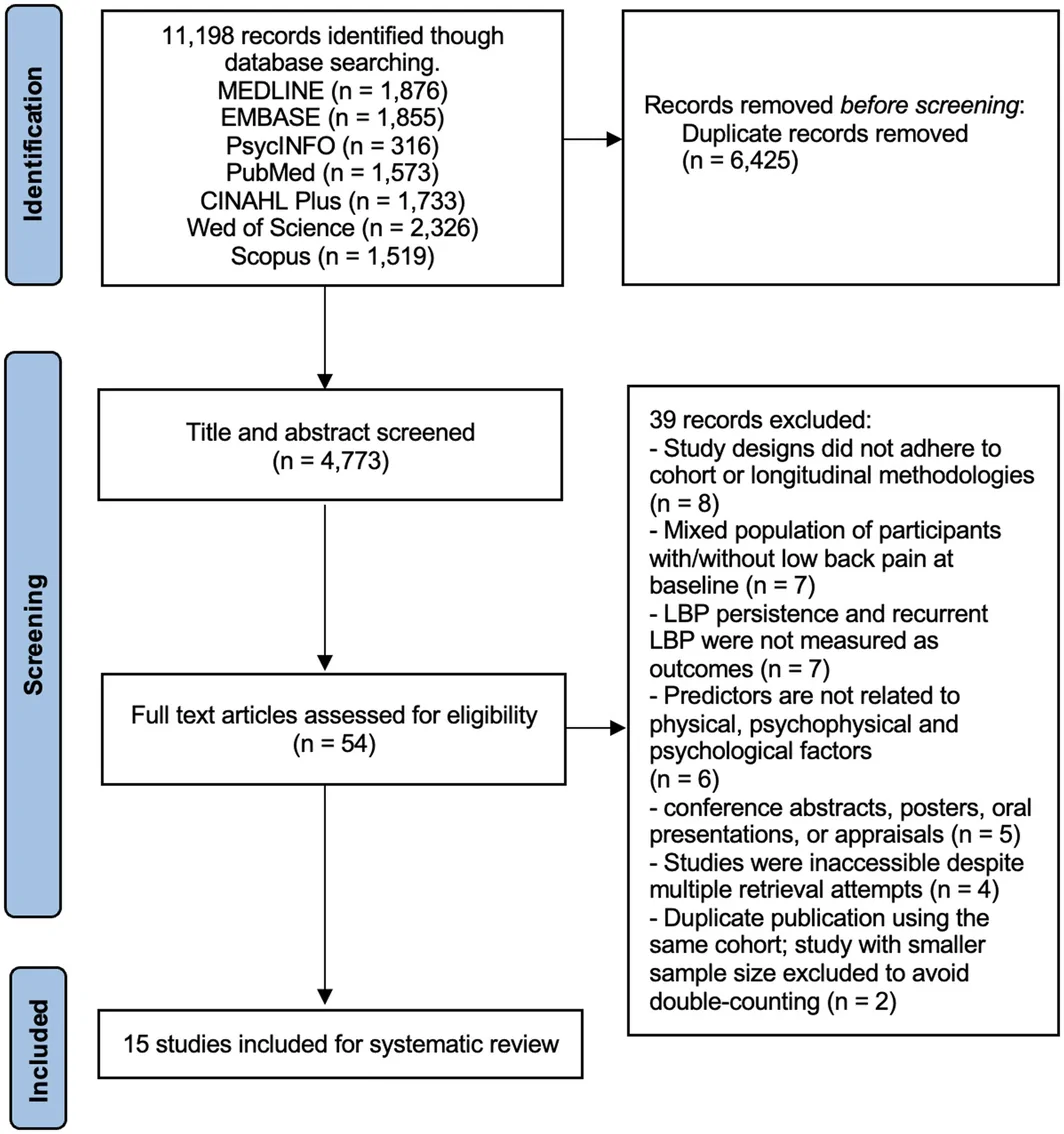
PRISMA flow diagram.

The study selection process followed a two‐stage approach. In the first stage, two reviewers (KW and GY) independently screened titles and abstracts of all retrieved records against predefined inclusion and exclusion criteria. In the second stage, the same two reviewers independently assessed full‐text articles of potentially eligible studies for final inclusion. Any disagreements between reviewers were resolved through discussion, and when consensus could not be reached, a third reviewer (DF) was consulted. Studies that met all inclusion criteria were included in the systematic review, and the selection process is illustrated in a PRISMA flow diagram (Figure 1). Reasons for exclusion of full‐text articles were recorded and are reported. Inter‐rater agreement for each stage of the study selection process was calculated using kappa statistics with IBM SPSS Statistics, Version 29.0 (2023). Kappa coefficient values were interpreted as follows: 0 indicates poor agreement; 0.01–0.20, slight agreement; 0.21–0.40, fair agreement; 0.41–0.60, moderate agreement; 0.61–0.80, substantial agreement; and 0.81–0.99, almost perfect agreement (McHugh 2012).

### 2.5 Data Items

Data extraction was conducted using the Checklist for critical Appraisal and data extraction for systematic Reviews of prediction Modelling Studies (CHARMS) (Moons et al. 2014). Although originally developed for validation studies of primary prediction models, this tool can be adapted for systematic reviews examining predictive factors through selective application of relevant domain items (Alalawi et al. 2019). Extracted data elements encompassed: (1) publication details (authors and year), (2) study location, (3) study methodology, (4) participant characteristics, (5) sample sizes, (6) physical, psychophysical or psychological predictive variables, (7) target outcomes, (8) follow‐up duration, (9) key findings, and (10) statistical analytical approaches.

### 2.6 Risk of Bias

The methodological quality of the included studies was assessed using the Quality in Prognosis Studies (QUIPS) tool (Table S2), which evaluated six domains: study participation, study attrition, measurement of predictive factors, control of confounding variables, outcome measurement and statistical analysis and reporting. The QUIPS tools has been validated for use in both prognostic and predictive factor studies (Hayden et al. 2013) and has been applied before in similar systematic reviews investigating predictive factors (Barbier et al. 2025; Yu et al. 2025). Two reviewers (KW and GY) independently assessed each study across six domains. Each domain evaluates the reporting of specific important information, with each element classified as ‘yes’, ‘no’, ‘partial’ or ‘unsure’. The risk of bias for each domain was then categorized as ‘high’, ‘moderate’ or ‘low’. A domain was rated as high risk if any element was rated ‘no’, and low risk if all elements were rated ‘yes’. Any combination of ‘yes’ with ‘partial’ or ‘unsure’ ratings was classified as moderate risk. An overall risk of bias rating (low, moderate or high) was assigned to each study based on the six domain assessments. Studies were classified as high overall risk if any domain was rated high risk, low overall risk if all domains were rated low risk, and moderate overall risk if domains showed a mix of low and moderate ratings with no high‐risk domains. Discrepancies between reviewers were resolved through discussion and consensus, ensuring consistency and accuracy in the quality assessment process. When disagreements persisted, a third reviewer (DF) was consulted for resolution.

### 2.7 Effect Measures and Synthesis of Results

Beta coefficients were used as the principal effect measure throughout the meta‐analysis. Effect estimates from individual studies required transformation to derive beta coefficients with appropriate distributional properties for statistical synthesis. Risk ratios (RR) were first converted to odds ratios (OR) using the equation below (Bonett 2007), where p0 equals the baseline risk (percentage incidence in the unexposed or reference group):

$$\mathrm{RR}=\frac{\text{OR}}{(1-p0+\left(p0\times \text{OR})\right)\;}$$

Subsequently, both converted OR and original OR from individual studies were transformed using natural logarithms [ln(OR)] to derive beta coefficients. Confidence intervals were similarly log‐transformed to maintain appropriate statistical properties for the meta‐analysis.

Meta‐analysis using random effects modelling performed with RStudio version 4.4.1 (2024), was conducted on studies with appropriate data for pooling. This modelling approach (Hedges et al. 2010) was adopted to address anticipated between‐study heterogeneity stemming from variations in study populations, research methodologies and clinical settings. The criterion for meta‐analysis inclusion required a minimum of 2 studies providing comparable outcome data (Deeks et al. 2024). Pooled effect estimates were computed by applying a weighted average of the beta coefficients from individual studies, where weights were assigned based on the inverse of the variance associated with each study's effect estimate. Heterogeneity across studies was evaluated using both the *I* ^2^ statistic and Q‐statistics to quantify and test for inconsistency in effect sizes. The *I* ^2^ statistic generated values between 0% and 100%, with heterogeneity levels classified as low (0%–30%), moderate (30%–50%), considerable (50%–70%) and substantial (70%–100%) (Cumpston et al. 2019). Meta‐analytic results were visualized using forest plots, which displayed individual study effect sizes along with their corresponding 95% confidence intervals, as well as the overall pooled estimate. Forest plots illustrated the magnitude and direction of associations between physical, psychophysical or psychological predictors and outcomes (persistent pain or recurrence in non‐specific LBP). Higher absolute values represented stronger associations, with positive coefficients indicating increased risk of persistent pain/recurrence and negative coefficients suggesting protective effects. However, for physical, psychophysical or psychological predictors reported by only one study, and for studies reporting different summary statistics (e.g., Spearman's rho correlation coefficients), narrative synthesis was conducted, as meta‐analysis was not feasible.

### 2.8 Certainty of Evidence

The overall certainty of evidence was assessed using the adapted GRADE (Grades of Recommendation, Assessment, Development and Evaluation) approach (Huguet et al. 2013) which provides a systematic framework for rating the certainty of evidence in systematic reviews and meta‐analyses. This approach classifies studies into three investigational phases based on their methodological rigour. Phase 1 studies involve preliminary research exploring possible relationships between prognostic factors and clinical outcomes. Phase 2 studies constitute confirmatory investigations that validate specific factor‐outcome associations, while Phase 3 studies examine causal pathways and mechanistic processes using comprehensive theoretical models. Evidence quality is initially rated as “high” for meta‐analyses from Phase 2 or 3 studies, reflecting their rigorous design and greater generalizability, whereas Phase 1 studies began at a “moderate” level due to their exploratory nature and typically smaller sample sizes. The quality rating for each comparison could then be downgraded by one or two levels based on five key criteria: (1) study limitations, if most included studies exhibited a moderate or high risk of bias as determined by the QUIPS tool; (2) inconsistency, if substantial heterogeneity was present (*I* ^2^ > 50%); (3) indirectness, if the population, prognostic factor or outcome did not align closely with the review question; (4) imprecision, when confidence intervals crossed zero while including meaningful beta coefficients in both directions, or when studies lacked adequate statistical power (below recommended thresholds of 10 outcome events per variable for dichotomous outcomes or 100 cases for continuous endpoints) to draw firm conclusions; and (5) publication bias, unless the prognostic factor had been extensively studied across multiple independent sources. Conversely, the quality rating could be upgraded by one or two levels in the presence of moderate to large effect sizes (moderate to large effect sizes defined as standardized beta coefficients of approximately 0.3 or greater for moderate effects and 0.5 or greater for large effects) (Cohen et al. 2003) or when a clear exposure–response gradient was observed (demonstration of progressive effect increases corresponding to elevated prognostic factor levels). Ultimately, the body of evidence was categorized into one of four certainty levels: high, moderate, low or very low, following the framework proposed by Huguet et al. (2013).

## 3 Results

### 3.1 Study Selection

The electronic search retrieved 11,198 records from multiple databases. Following duplicate removal (*n* = 6425), 4773 records underwent title and abstract screening, with an almost perfect inter‐reviewer agreement (*k* = 0.92). From these, 54 studies were proceeded to full‐text evaluation (Figure 1). During the comprehensive full‐text assessment, 39 studies were excluded for methodological or content‐related reasons. The primary reasons for exclusion were: study designs that did not meet cohort or longitudinal criteria (*n* = 8); inclusion of a mixed population of participants with and without low back pain at baseline (*n* = 7); absence of outcomes related to LBP persistence or recurrence (*n* = 7); predictors not related to physical, psychophysical or psychological factors (*n* = 6); conference abstracts, posters, oral presentations or appraisals (*n* = 5); full‐text not accessible (*n* = 4); and duplicate publications using the same cohort, with the smaller sample excluded to avoid double counting (*n* = 2). A detailed list of all excluded studies along with the specific reason for exclusion is provided in Table S3. The agreement for full‐text screening was also almost perfect (*k* = 0.86). In total, 15 studies successfully met all established eligibility criteria and were included in the final systematic review for analysis. All studies focused on predicting persistent pain and not a single study was identified which examined predictors of recurrent LBP (which was defined as a new episode of LBP following a pain‐free period of at least 1 month).

### 3.2 Study Characteristics

The characteristics of included cohort and longitudinal studies are presented in Table 1. Across all 15 studies (total baseline *n* = 15,522; follow‐up *n* = 14,119), the mean age of participants was 59.2 years (14 studies reporting age data), with 61.7% being female participants (15 studies reported gender data). All studies examined persistent LBP, while no studies included participants with recurrent LBP at follow up. Sample sizes ranged from 58 to 5740 participants at baseline and from 45 to 5740 participants at follow‐up.

**TABLE 1:** Overview of key characteristics from the included studies.

Author, year	Study design	Population at baseline and participant characteristics	Follow‐up period	Potential physical, psychophysical and psychological predictors	Outcomes	Population at follow‐up	Statistical analysis	Findings
Basinski et al. (2022)	Prospective cohort study	*n* = 110—Non‐specific acute LBP at baseline—Mean age 39.3 (11.1) years—Male 54.1%—Mean pain intensity 6.02 (1.85)/10—Mean disability (ODI) 22.61 (11.85)/50	3 months	Psychological predictors: Anxiety and depression symptoms (HADS) Strategies of coping with pain (CSQ)	Presence of persistent LBP (yes/no)	*n* = 97	Multiple logistic regression	Anxiety: OR = 1.05 (0.86–1.30) Depression: OR = 0.96 (0.77–1.18) Catastrophising: OR = 0.97 (0.87–1.07) CSQ, Distraction: OR = 1.07 (0.95–1.22) CSQ, Ignoring sensations: OR = 1.08 (0.97–1.22) CSQ, Praying and hoping: OR = 0.93 (0.85–1.02)
Dunn and Croft (2006)	Prospective cohort study	*n* = 359—cLBP at baseline—Mean age 46.9 (8.3) years—Female 58.2%—Mean pain intensity 4.6 (2.6)/10—Mean disability (RMDQ) 10.3 (6.6)/24	12 months	Psychological predictors: Fear avoidance (TSK) Catastrophising (CSQ)	CPG IV	*n* = 346	RR	Fear avoidance: RR = 2.4 (1.6, 3.7)* Catastrophising: RR = 5.5 (3.5–8.9)*
Ferguson and Marras (2013)	Prospective study	*n* = 206—LBP at baseline—Mean age 41.8 (10.3) years—Male 73%—Mean pain intensity 50.7 (27.1)/100—Mean disability (RMDQ) 10.3 (6.6)/24	12 months	Physical predictors: Sagittal range of motion Sagittal flexion/extension velocity Sagittal flexion/extension acceleration	Presence of LBP at follow up	*n* = 196	Logistic regression analysis	Sagittal range of motion: OR = 3.09 (0.84–11.35) Sagittal flexion/extension velocity: OR = 2.18 (1.21–3.94)*/OR = 2.03 (1.11–3.70)* Sagittal flexion/extension acceleration: OR = 2.31 (1.22–4.41)*/OR = 5.14 (1.13–23.45)*
Gomes et al. (2023)	Cohort study	*N* = 1201—cLBP at baseline—Mean age 61.6 (13.6) years—Female 78.4%—Mean disability (HAQ) 0.99 (0.72)/3—Mean HRQoL (EQ‐5D‐3L) 0.46 (0.19)/1	Three time‐points over 5 years	Psychological predictors: Anxiety and depression (HADS)	Presence of persistent LBP	*n* = 400	Binary logistic regression analysis	Depressive positive symptoms: OR = 1.96 (1.21–3.18)*
Halonen et al. (2019)	Prospective longitudinal cohort study	*n* = 5740—LBP at baseline—Mean age of the total study population 54.1 (11.3) years—Female 61%—BMI: Normal 44%, Overweight 39%, Obese 17%	Every 2 years for 6 years	Psychological predictors: Depression symptoms (SCL‐CD6)	Presence of persistent LBP	*n* = 9653 (number of observations)	Binomial regression	Depressive symptoms: RR = 1.09 (1.05–1.14)*
Hendrick et al. (2013)	Prospective cohort study	*n* = 101—Acute LBP (≤ 6 weeks) at baseline—Mean age 37.8 (14.6) years—Female 50.5%—Mean pain intensity 57.4 (19.7)/100—Mean disability (RMDQ) 8.1 (3.8)/24	3 months	Psychological predictors: Psychological distress (GHQ12) Fear avoidance (FABQPA)	Disability (RMDQ)	*n* = 90	Multiple linear regression	Psychological distress: *β* = 0.05 (−0.12–0.58) FABQPA: *β* = 0.09 (−0.03–0.14)
Jones et al. (2006)	Prospective cohort study	*n* = 974—LBP at baseline—Median age 47 (38, 56) years—Female 59%—Median pain intensity 30 (12–50)/100—Median disability (RMDQ) 8 (4–13)/24	3 months	Psychological predictors: Passive and active coping (VPMI)	Persistent LBP defined by VAS ≥ 20 mm and RMDQ ≥ 5	*n* = 922	Poisson regression	Passive coping: RR = 1.4 (1.01–1.9)
Klyne et al. (2022)	Prospective cohort study	*n* = 133—Acute LBP at baseline—Mean age 29 (7.9) years—Female 50.8%—Mean BMI 24.3 (4.0) kg/m^2^—Mean Pain (NRS) 5.0 (1.8)/10—Mean Disability (RMDQ) 6.8 (4.4)/28	12 months (with assessments every 2 weeks for pain, and at 3, 6, 9, 12 months for comprehensive measures)	Psychological predictors: Depression symptoms (CES‐D) Pain catastrophizing (PCS) Fear‐avoidance, work (FABQ‐W) Fear‐avoidance, physical activity (FABQ‐PA) Pain self‐efficacy (PSEQ)	LBP intensity (NRS) and Disability (RMDQ)	3 months: *n* = 120 6 months: *n* = 97 9 months: *n* = 85 12 months: *n* = 94	GEE	1. Depression: 6 months, not retained in final model 9 months, Coefficient 0.01 (SE 0.00), *R* ^2^ = 0.06* 12 month: Coefficient 0.01 (SE 0.00), *R* ^2^ = 0.10* 2. Catastrophizing: 6 months, Coefficient 0.02 (SE 0.01), *R* ^2^ = 0.10*, 9 months, Coefficient 0.02 (SE 0.00), *R* ^2^ = 0.09* 12 months, Coefficient 0.01 (SE 0.00), *R* ^2^ = 0.07* 3. Fear Avoidance—Work (FABQ‐W): 6 months, Coefficient 0.02 (SE 0.00), *R* ^2^ = 0.13* 9 months, Coefficient 0.02 (SE 0.00), *R* ^2^ = 0.12* 12 months, Not retained in final model 4. Fear avoidance—Physical Activity (FABQ‐PA): 6 months, Coefficient 0.01 (SE 0.01), *R* ^2^ = 0.03, not significant 9 months, Coefficient 0.00 (SE 0.01), *R* ^2^ = 0.00, not significant 12 months, Not retained in final model 5. Pain self‐efficacy: 6 months, Coefficient −0.01 (SE 0.00), *R* ^2^ = 0.18* 9 months, not retained in final model 12 months, Coefficient −0.02 (SE 0.00), *R* ^2^ = 0.22*
Leresche et al. (2013)	Prospective cohort study	*n* = 157—LBP at baseline—Mean age 47.4 (12.4) years—Female 61.8%	4 months	Psychophysical and psychological predictors: PPT CPP CPM mTS Depression (PHQ‐8), anxiety (GAD‐2)	Clinically significant back pain (GCP, Grades II–IV)	*n* = 147	Logistic regression	PPT, back: OR = 0.72 (0.46–1.11) PPT, thenar eminence: OR = 0.70 (0.44–1.06) Conditioned pain modulation: OR = 1.07 (0.73–1.56) Mechanical temporal summation: OR = 0.88 (0.58–1.27) Cold pressor pain: OR = 0.91 (0.61–1.36)
Melloh et al. (2013)	Prospective cohort study	*n* = 315—LBP < 12 weeks at baseline—Mean age 34.9 (12.6) years—Mean BMI 28 (6) kg/m^2^—Female 66%	6 months	Psychological predictors: Depression (Zung SDS) Somatization (MSPQ) Fear‐avoidance (FABQ) Catastrophizing (PCS)	Persistent LBP defined by VAS ≥ 20 mm at baseline and 6 months with < 20 mm change	*n* = 168	Multiple logistic regression analysis	Depression: OR = 1.03 (0.98–1.08) FABQ‐W: OR = 1.01 (0.97–1.05) FABQPA: OR = 1.02 (0.95–1.11) Catastrophizing: OR = 0.99 (0.95–1.04)
Petersen et al. (2020)	Prospective cohort study	*n* = 58—LBP at baseline—Mean age 44.8 (16.7) years—Mean BMI 29.1 (6.2) kg/m^2^—Female 51.1%	12 weeks	Psychophysical and psychological predictors: TSP Catastrophizing (PCS)	LBP intensity (VAS)	*n* = 45	Linear regression	TSP effect: *β* = 0.31, PCS: *β* = 0.49
Rundell et al. (2017)	Prospective cohort study	*n* = 5220—LBP at baseline—Mean age 73.8 (6.9) years—Female 64.7%—Mean pain intensity (NRS) 5.0 (2.8)/10—Mean disability (RMDQ) 9.6 (6.4)/24	6 months and 12 months	Psychological predictors: Depressive and anxiety (PHQ‐4)	Persistent LBP at 6 and 12 months (defined by NRS ≥ 3/10 at both time points)	*n* = 5206	Logistic regression	Anxiety: OR = 1.65 (1.33–2.03)* Depression: OR = 2.10 (1.60–2.75)*
Sieben et al. (2005)	Prospective cohort study	*n* = 222—Acute LBP < 3 weeks at baseline—Age 45–54 years 38.7%—Male 56.3%	3, 6, 12 months and 3 years	Psychological predictors: Catastrophizing (PCS) Fear avoidance (TSK) Depression (BDI)	GCPS	*n* = 167	Spearman rank correlation coefficients	Catastrophising: Spearman's *r* correlation coefficients = 0.189* Fear avoidance: Spearman's *r* correlation coefficients = 0.175* Depression: Spearman's *r* correlation coefficients = 0.265*
Swinkels‐Meewisse et al. (2006)	Prospective cohort study	*n* = 555—Acute LBP < 4 weeks at baseline—Mean age 43.3 years—Male 58%	6 weeks and 6 months	Psychological predictors: Fear avoidance (TSK)	LBP disability (RMDQ)	*n* = 337	Multilevel analyses	Fear avoidance: *β* = 0.23*
van der Windt et al. (2007)	Prospective cohort study	*n* = 171—LBP < 13 weeks at baseline—Mean age 42 (12) years—Male 52.6% ‐ Mean pain 4.8 (1.9)/10 ‐ Mean disability (RMDQ) score 50.8 (20.7)/100	3 months	Psychological predictors: Catastrophising (PCCL) Coping Strategies (CSQ) Fear‐avoidance (FABQ) Distress and somatisation (4DSQ)	Persistent LBP at 3 months defined as not reporting full recovery or very large improvement on 7‐point Likert scale	*n* = 164	Logistic regression analysis	Catastrophising: OR = 2.45 (1.09–5.51)* Somatisation: OR = 2.14 (0.90–5.08) Distress: OR = 1.86 (0.86–4.05)

Abbreviations: 4DSQ, Four‐Dimensional Symptom Questionnaire; BDI, Beck Depression Inventory; BMI, body mass index; CES‐D, Center for Epidemiological Studies Depression Scale; CPG, Chronic Pain Grade; CPM, conditioned pain modulation; CPP, cold pressor pain; CSQ, Coping Strategies Questionnaire; FABQ, Fear‐Avoidance Beliefs; FABQPA, Fear‐Avoidance Beliefs Questionnaire for physical activity; FABQ‐W, Fear‐Avoidance Beliefs Questionnaire—Work subscale; GAD‐2, Generalized Anxiety Disorder‐2; GCPS, Graded Chronic Pain Scale; GEE, Generalized Estimating Equations; GHQ, General Health Questionnaire; GHQ12, 12‐item General Health Questionnaire; HADS, Hospital Anxiety and Depression Scale; HAQ, Health Assessment Questionnaire; LBP, Low Back Pain; MSPQ, Modified Somatic Perception Questionnaire; mTS, Mechanical Temporal Summation; NRS, Numeric Rating Scale; ODI, Oswestry Disability Index; OR, Odds Ratio; PCCL, Pain Coping and Cognition List; PCS, Pain Catastrophizing Scale; PHQ‐4, Patient Health Questionnaire‐4; PHQ‐8, Patient Health Questionnaire‐8; PPT, Pressure Pain Thresholds; PSEQ, Pain Self‐Efficacy Questionnaire; rLBP, Recurrent Low Back Pain; RMDQ, Roland‐Morris Disability Questionnaire; RR, relative risk; SCL‐CD6, Symptom Checklist‐core depression scale, six‐item questionnaire; SE, standard error; TSK, Tampa Scale for Kinesiophobia; TSP, temporal summation of pain; VAS, Visual Analogue Scale; VPMI, Vanderbilt Pain Management Inventory; Zung SDS, Zung Self‐Rating Depression Scale.

* Significant findings.

Physical, psychophysical and psychological predictors are detailed in Table 1. Regarding outcome measures, only seven studies reported the primary outcome of LBP persistence, measured by the presence of pain or disability evaluated at different time points during the follow‐up period, or according to each study's specific definition of persistent LBP. Back pain intensity and disability levels were reported in eight studies. Follow‐up periods across all studies ranged from 3 months to 6 years. Eight studies reported short‐term follow‐up (≤ 6 months), while seven studies reported long‐term follow‐up (> 6 months).

### 3.3 Risk of Bias of Individual Studies

The risk of bias evaluation for individual studies is presented in Table 2. The majority of studies (*n* = 10) demonstrated high risk of bias, while four studies showed moderate risk and only one study exhibited low risk of bias. High risk of bias was attributed to substantial limitations identified across three methodological domains. Study attrition issues were evident, with poor documentation of participant tracking procedures and insufficient justification for study dropouts. Most studies showed confounding measurement and account problems, with insufficient adjustment for crucial variables including initial pain measurements and co‐existing conditions, along with inappropriate management of incomplete confounding data. Predictive factor measurement deficiencies were found in eight studies that demonstrated substandard handling of missing predictive factor data through inadequate imputation approaches. Inter‐reviewer agreement for bias assessment reached 83.33%. Most discrepancies between reviewers occurred in domain 3 (predictive factor measurement) and domain 5 (study confounding). All reviewer discrepancies were successfully resolved through discussion.

**TABLE 2:** Risk of bias assessment using the Quality in Prognosis Studies (QUIPS) tool for studies exploring physical, psychophysical and psychological factors predicting persistent non‐specific low back pain.

References	Study participation	Study attrition	Prognostic factor measurement	Outcome measurement	Confounding measurement and account	Analysis and reporting	Overall risk of bias
Basinski et al. (2022)							
Dunn and Croft (2006)							
Ferguson and Marras (2013)							
Gomes et al. (2023)							
Halonen et al. (2019)							
Hendrick et al. (2013)							
Jones et al. (2006)							
Klyne et al. (2022)							
Leresche et al. (2013)							
Melloh et al. (2013)							
Petersen et al. (2020)							
Rundell et al. (2017)							
Sieben et al. (2005)							
Swinkels‐Meewisse et al. (2006)							
van der Windt et al. (2007)							

*Note:* 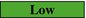 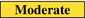 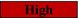.

### 3.4 Data Synthesis and Meta‐Analysis

Meta‐analysis was performed when a minimum of two studies provided effect sizes related to persistent or recurrent non‐specific LBP. Since only a single study examined trunk movement kinematics as a physical predictor, this finding is presented via a narrative synthesis. Similarly, as only a single study examined conditioned pain modulation and pressure pain thresholds as psychophysical predictors, these findings are also presented via a narrative synthesis.

### 3.5 Physical Predictors of Persistent LBP

One physical predictor was examined: trunk kinematics (trunk sagittal range of motion, sagittal trunk flexion/extension velocity, sagittal trunk flexion/extension acceleration), which was analysed narratively due to insufficient data for meta‐analysis.

#### 3.5.1 Trunk Kinematics

One study examined trunk kinematic features as predictors of persistent LBP. Ferguson and Marras (2013) assessed 206 workers at baseline using Lumbar Motion Monitor and followed them for 1 year (Ferguson and Marras 2013). They found that during controlled sagittal bending, trunk flexion was not statistically significant for predicting persistent LBP. However, slower flexion velocity (≤ 38.0°/s; OR 2.18, 95% CI 1.21–3.94) and extension velocity (≤ 40.0°/s; OR 2.03, 95% CI 1.11–3.70) each approximately double the odds, while severely limited extension acceleration (≤ 64.8°/s^2^) increased the odds more than five‐fold (OR 5.14, 95% CI 1.13–23.45). The certainty of this evidence is very low due to this being a single study.

### 3.6 Psychophysical Predictors of Persistent LBP

Several psychophysical predictors derived from quantitative sensory testing were examined, including temporal summation of pain, pressure pain thresholds, conditioned pain modulation and cold pressor response. Among these, only temporal summation of pain provided sufficient data for meta‐analysis, while the remaining measures were analysed narratively.

#### 3.6.1 Pain Sensitivity (Temporal Summation of Pain, Pressure Pain Thresholds, Conditioned Pain Modulation, Cold Pressor Pain)

**FIGURE 2:**
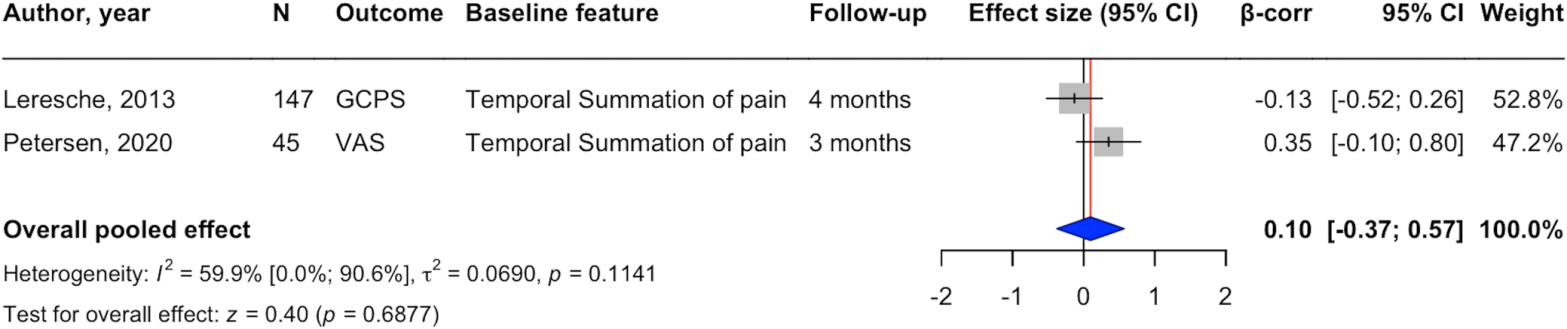
Meta‐analysis of temporal summation of pain as a predictor of LBP.

Two studies (Leresche et al. 2013; Petersen et al. 2020) (*n* = 192) investigated temporal summation of pain as a predictive factor for pain outcomes over 3–4‐month follow‐up intervals. The outcome of pain outcomes was measured using the VAS and the Graded Chronic Pain Scale (GCPS). Enhanced temporal summation of pain was not a significant predictor of persistent LBP (*β* = 0.10, 95% CI: −0.37 to 0.57) (Figure 2). Given the limited evidence from only two studies, the certainty of these findings remains very low. Other pain sensitivity measures showed limited predictive value for persistent LBP. Leresche et al. (2013) found that lower pressure pain thresholds (indicating higher pain sensitivity) assessed over the back and at distant sites predicted persistent LBP at a 4 month follow‐up; however, the certainty of evidence was very low (Leresche et al. 2013). Central pain modulation measures including conditioned pain modulation and cold pressor responses were not significant predictors of persistent LBP (Leresche et al. 2013).

### 3.7 Psychological Predictors of Persistent LBP

Seven psychophysical predictors were examined: depression and psychological distress, anxiety, fear avoidance, catastrophising, somatisation, pain coping strategy and pain self‐efficacy. Depression and psychological distress, anxiety, fear avoidance, catastrophising, somatisation and pain coping strategy were assessed via meta‐analysis. Pain self‐efficacy was examined narratively, as only one study met the inclusion criteria for this factor.

#### 3.7.1 Depression and Psychological Distress

Regarding depression and psychological distress, five studies examined their predictive value for persistent LBP and disability, and two studies examined psychological distress as a predictor of both persistent LBP and disability (Hendrick et al. 2013; van der Windt et al. 2007). Five studies reported depression as a predictor of persistent LBP (Basinski et al. 2022; Gomes et al. 2023; Halonen et al. 2019; Melloh et al. 2013; Rundell et al. 2017). Depression was measured using the Hospital Anxiety and Depression Scale (HADS), Symptom Checklist Depression scale (SCL‐CD), Zung Self‐Rating Depression Scale (Zung SDS) and Patient Health Questionnaire‐4 Depression and Anxiety Screen (PHQ‐4), with outcomes assessed as persistent LBP. Psychological distress was measured by the General Health Questionnaire (GHQ) and Four‐Dimensional Symptoms Questionnaire (4DSQ), with outcomes assessed through RMDQ scores and persistent LBP. Depression and psychological distress were grouped together as they share a common underlying dimension of negative affectivity (Henry and Crawford 2005), with depression conceptualized as part of general psychological distress (Lee et al. 2015) and both constructs having been treated as related factors in previous systematic reviews of predictors of LBP (Pincus et al. 2002).

**FIGURE 3:**
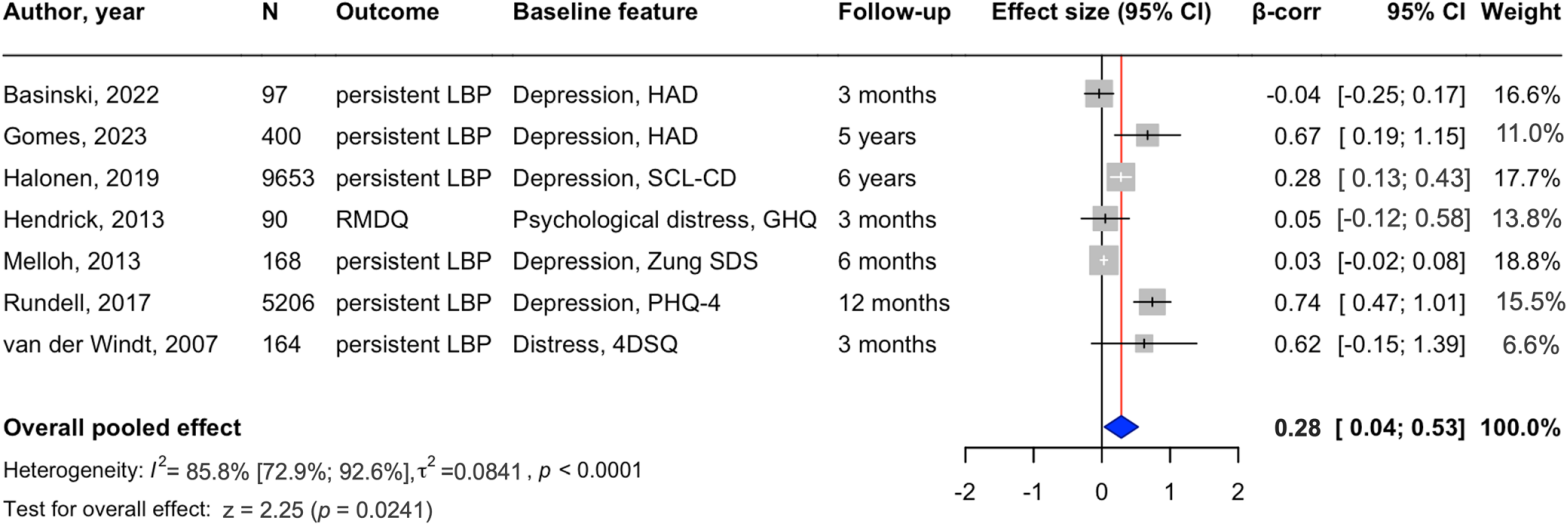
Meta‐analysis of depression and distress merged as a predictor of persistent LBP and disability outcomes.

Meta‐analysis of seven studies (Basinski et al. 2022; Gomes et al. 2023; Halonen et al. 2019; Hendrick et al. 2013; Melloh et al. 2013; Rundell et al. 2017; van der Windt et al. 2007) demonstrated that depression and distress significantly predicted persistent LBP and disability (*n* = 15,778; *β* = 0.28, 95% CI: 0.04 to 0.53, *p* = 0.02) (Figure 3). However, substantial between‐study heterogeneity was observed (*I* ^2^ = 85.8%, *p* < 0.01), indicating considerable variability in effect estimates across different study populations and methodologies. GRADE evaluation revealed low certainty of evidence regarding the predictive ability of depression and distress for persistent LBP and disability at short‐ and long‐term follow‐up. These findings should therefore be interpreted with caution, given the high risk of bias identified across most included studies.

Two studies with incompatible effect measures underwent narrative synthesis. Depression exhibited varying predictive patterns between studies. Sieben et al. (2005) identified that depressive symptoms (negative self‐perception) achieved statistical significance only at extended follow‐up intervals (6–12 months), with correlation coefficients ranging from 0.165 to 0.265 (Sieben et al. 2005). Klyne et al. (2022) reported that depressive symptoms at baseline consistently predicted worse outcomes at 3, 6 and 12 months (Klyne et al. 2022).

#### 3.7.2 Anxiety

**FIGURE 4:**
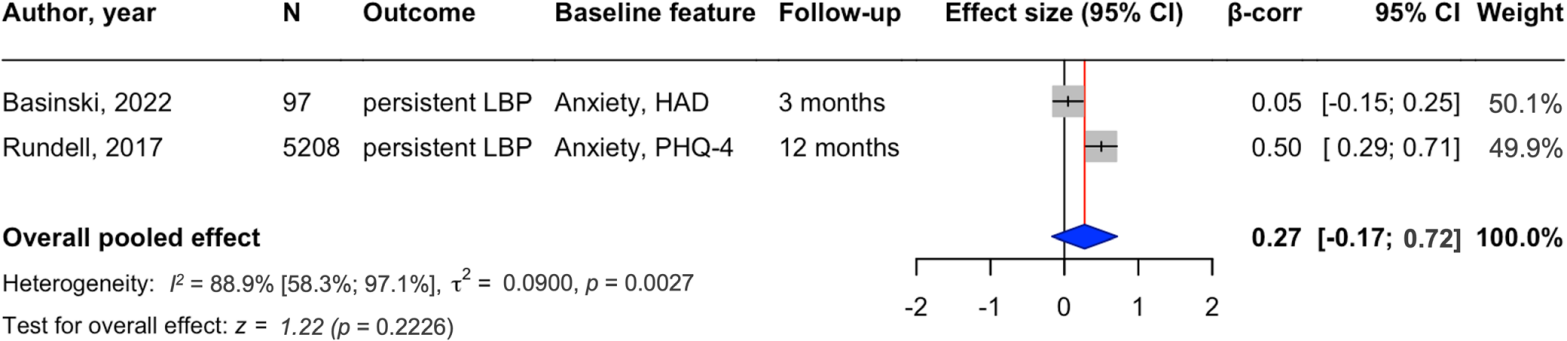
Meta‐analysis of anxiety as a predictor of persistent LBP outcomes.

Two studies (*n* = 5305) investigated anxiety as a predictor of persistent LBP, with follow‐up periods ranging from 3 to 12 months (Basinski et al. 2022; Rundell et al. 2017). Anxiety was measured using the HADS and PHQ‐4. Meta‐analysis revealed that anxiety was not significant predictor of persistent LBP (*β* = 0.27, 95% CI: −0.17 to 0.72, *p* = 0.22), although substantial heterogeneity was observed (*I* ^2^ = 88.9%, *p* < 0.01) (Figure 4). GRADE evaluation showed low certainty of evidence for the predictive value of anxiety for persistent LBP.

#### 3.7.3 Fear Avoidance

**FIGURE 5:**
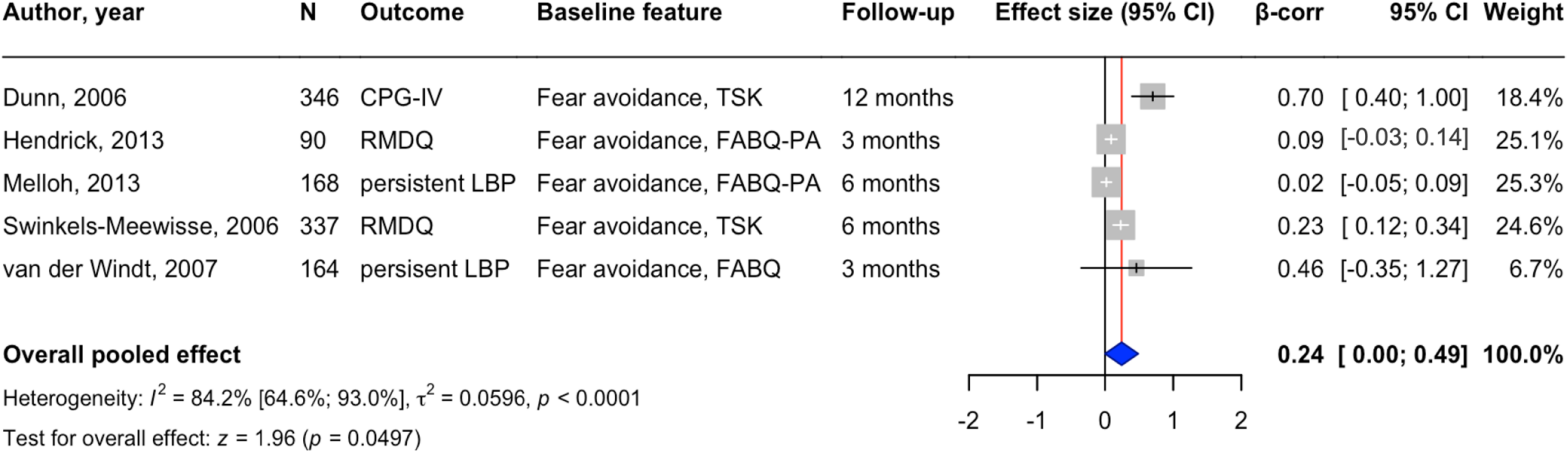
Meta‐analysis of fear avoidance as a predictor of persistent LBP and disability outcomes.

Five studies (*n* = 1105) investigated fear avoidance as a predictor of LBP‐related outcomes, with follow‐up intervals ranging from 3 months to 1 year (Dunn and Croft 2006; Hendrick et al. 2013; Melloh et al. 2013; Swinkels‐Meewisse et al. 2006; van der Windt et al. 2007). Outcomes included persistent LBP, LBP episodes, pain‐related disability (CPG‐IV) and functional disability (RMDQ). Fear avoidance was assessed using the Fear Avoidance Beliefs Questionnaire (FABQ) and Tampa Scale for Kinesiophobia (TSK). The meta‐analysis revealed that fear avoidance demonstrated a small positive predictive effect on persistent LBP (*β* = 0.24, 95% CI: 0.00 to 0.49, *p* = 0.049), although substantial heterogeneity was observed (*I* ^2^ = 84.2%, *p* < 0.01) (Figure 5). However, statistical significance was borderline, and this finding should be interpreted cautiously. GRADE evaluation revealed low certainty of evidence regarding the predictive value of fear avoidance for persistent LBP. These findings should therefore be interpreted with caution, given the high risk of bias identified across most included studies. Two additional studies were included in the narrative synthesis due to incompatible effect measures but provided relevant insights. Sieben et al. (Sieben et al. 2005) found that activity avoidance fear showed weak‐to‐moderate predictive factors for disability outcomes at baseline and study completion, but did not significantly predict outcomes at intermediate follow‐up periods. Conversely, Klyne et al. (Klyne et al. 2022) demonstrated that work‐related fear avoidance predicted elevated LBP intensity at a 9‐month follow‐up. These contrasting findings suggest that different fear‐avoidance constructs or study populations may yield varying predictive relationships.

#### 3.7.4 Catastrophising

**FIGURE 6:**
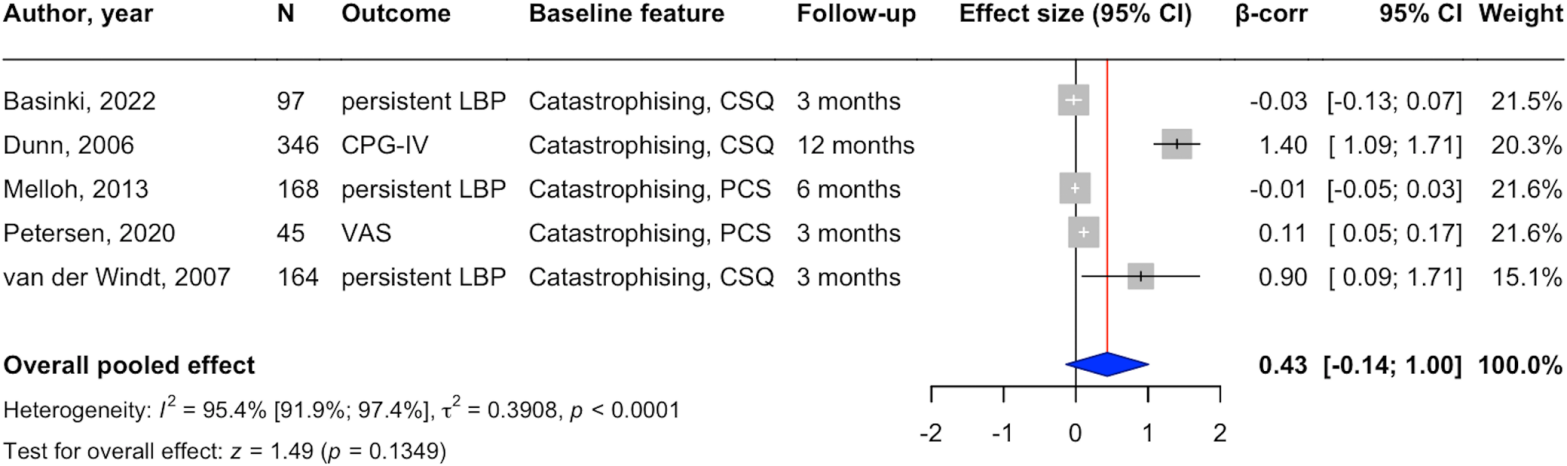
Meta‐analysis of catastrophising as a predictor of persistent LBP outcomes.

Five studies (*n* = 820) investigated catastrophizing as a predictor of persistent LBP with follow‐up periods ranging from 3 months to 12 months (Basinski et al. 2022; Dunn and Croft 2006; Melloh et al. 2013; Petersen et al. 2020; van der Windt et al. 2007). Outcomes included persistent LBP, Chronic Pain Grade (CPG) and VAS scores. Catastrophizing was assessed using the Coping Strategies Questionnaire (CSQ) and Pain Catastrophizing Scale (PCS). Meta‐analysis revealed that catastrophising was not a significant predictor of persistent LBP, CPG and VAS (*β* = 0.43, 95% CI: −0.14 to 1.00, *p* = 0.13), with considerable heterogeneity observed (*I* ^2^ = 95.4%, *p* < 0.01) (Figure 6). GRADE evaluation showed very low certainty of evidence for the predictive value of catastrophizing. Narrative synthesis included two studies excluded from meta‐analysis due to incompatible measures. Sieben et al. (2005) found that pain catastrophizing demonstrated weak but consistent predictive factors for disability outcomes across most follow‐up periods. Conversely, Klyne et al. (2022) demonstrated that the predictive capacity of pain catastrophizing varied depending on measurement timing, with baseline assessments predicting pain at 3, 6 and 9 months, while 3‐month measurements showed enhanced predictive relationships.

#### 3.7.5 Somatisation

**FIGURE 7:**
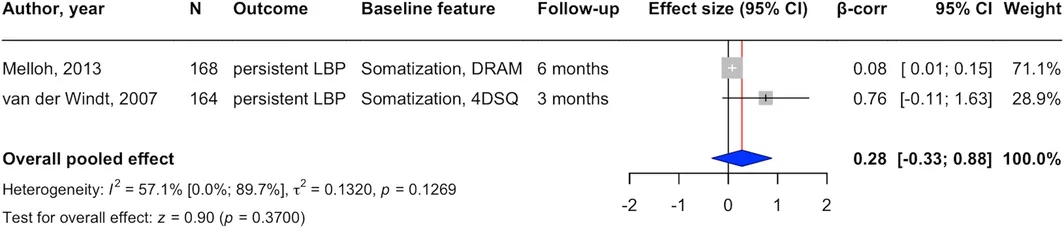
Meta‐analysis of somatization as a predictor of persistent LBP.

Two studies (*n* = 332) investigated somatisation as a predictor of persistent LBP, with follow‐up periods ranging from 3 to 6 months (Melloh et al. 2013; van der Windt et al. 2007). Somatisation was assessed using two questionnaires: the Distress and Risk Assessment Method (DRAM) and the Four‐Dimensional Symptom Questionnaire (4DSQ). Meta‐analysis revealed that somatisation was not a significant predictor of persistent LBP (*β* = 0.28, 95% CI: −0.33 to 0.88, *p* = 0.37), with moderate heterogeneity observed (*I* ^2^ = 57.1%, *p* = 0.13) (Figure 7). GRADE assessment indicated very low certainty of evidence for this predictive relationship.

#### 3.7.6 Pain Coping Strategy

**FIGURE 8:**
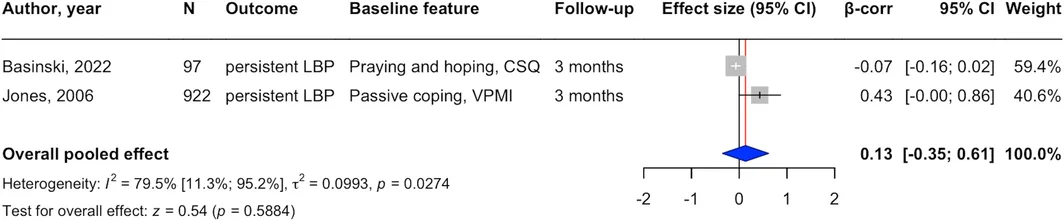
Meta‐analysis of passive coping as a predictor of persistent LBP.

Two studies (*n* = 1019) examined passive coping as a predictor of persistent LBP, with a 3‐month follow‐up (Basinski et al. 2022; Jones et al. 2006). Basinski et al. (2022) measured coping using the Coping Strategies Questionnaire (CSQ), while Jones et al. (2006) assessed pain coping strategies using the Vanderbilt Pain Management Inventory (VPMI). Jones et al. (Jones et al. 2006) categorized participants into active and passive coping groups, finding that passive coping strategies significantly predicted increased risk of persistent LBP. In contrast, Basinski et al. (2022) did not use active/passive categorisation; however, their passive coping measure comprised praying and hoping domains from the CSQ. Therefore, the meta‐analysis focused on passive coping constructs from both studies: praying and hoping and passive coping strategies. Meta‐analysis revealed that passive coping was not a significant predictor of persistent LBP (*β* = 0.13, 95% CI: −0.35 to 0.61, *p* = 0.59), with substantial heterogeneity observed (*I* ^2^ = 79.5%, *p* = 0.03) (Figure 8). GRADE analysis demonstrated very low certainty of this evidence regarding the predictive value of passive coping for persistent LBP.

#### 3.7.7 Pain Self‐Efficacy

One study examined pain self‐efficacy as predictors of persistent LBP. Klyne et al. (2022) assessed 133 individuals with acute LBP, measuring pain self‐efficacy at 3 months post‐onset using the Pain Self‐Eefficacy Questionnaire (PSEQ). Lower pain self‐efficacy at 3 months predicted higher pain levels at 6 and 12 months, with higher self‐efficacy predicting better pain outcome. However, pain self‐efficacy was not retained in the 9‐months model, suggesting its predictive factor may be inconsistent across time points. The certainty of this evidence is very low due to reliance on a single study.

### 3.8 Quality Assessment

According to the GRADE analysis (Table 3), evidence certainty was categorized into two levels: low and very low. Of the predictors assessed, three were rated as low certainty (depression/distress, anxiety and fear avoidance) and eight were rated as very low certainty (catastrophising, somatisation, passive coping and physical measures including temporal summation of pain, pressure pain thresholds, conditioned pain modulation, cold pressor pain and trunk movement kinematics).

**TABLE 3:** Overall certainty of evidence for physical, psychophysical and psychological predictors of persistent low back pain and disability.

Outcome: persistent low back pain and disability																	
Physical, psychophysical and psychological predictors	Number of participants	Number of studies	Univariate			Multivariate			GRADE factors								
			+	0	−	+	0	−	Phase	Study limitations	Inconsistency	Indirectness	Imprecision	Publication bias	Moderate/large size	Dose effect	Overall quality
Depression and distress	15,778	7	0	1	0	3	3	0	2	X	X	✓	✓	✓	✓	NA	++
Anxiety	5305	2	0	1	0	1	0	0	1	X	X	✓	✓	X	X	NA	++
Fear avoidance	1105	5	1	0	0	1	3	0	2	X	X	✓	X	✓	✓	NA	++
Catastrophising	820	5	1	1	0	2	1	0	2	X	X	✓	X	✓	✓	NA	+
Somatisation	332	2	0	0	0	1	1	0	2	X	X	✓	X	✓	✓	NA	+
Passive coping	1019	2	0	1	0	1	0	0	1	X	X	✓	X	✓	✓	NA	+
Pain self‐efficacy^a^	133	1	1	0	0	0	0	0	1	X	NA	NA	NA	NA	NA	NA	+
Temporal summation of pain	192	2	0	0	0	0	2	0	2	X	X	✓	X	X	X	NA	+
Pressure pain thresholds^a^	147	1	1	0	0	0	0	0	1	X	NA	NA	NA	NA	NA	NA	+
Conditioned pain modulation^a^	147	1	0	1	0	0	0	0	1	X	NA	NA	NA	NA	NA	NA	+
Cold pressor pain^a^	147	1	0	1	0	0	0	0	1	X	NA	NA	NA	NA	NA	NA	+
Trunk movement kinematics^a^	206	1	1	0	0	0	0	0	2	X	NA	NA	NA	NA	NA	NA	+

*Note:* Phase indicates the research investigation stage. Statistical results notation: +, significant positive predictor; 0, non‐significant predictor; −, significant negative predictor. Quality assessment symbols: ✓, no serious limitations; ✕, serious limitations (or absent for effect size/dose criteria); NA, unable to rate item based on available information. For overall quality of evidence: + (very low), ++ (low), +++ (moderate), ++++ (high).

^a^ For features reported in only one study, the overall certainty is graded very low due to serious concerns regarding imprecision, publication bias and lack of replication.

The primary factors leading to downgraded certainty of evidence included study limitations (most studies showed moderate to high risk of bias), imprecision (wide confidence intervals or small total sample sizes), and inconsistent results (high heterogeneity). Notably, each of the physical predictors were reported in only one study; therefore, the overall certainty was graded as very low due to serious concerns regarding imprecision, publication bias, and lack of replication.

## 4 Discussion

This systematic review and meta‐analysis synthesized current evidence regarding physical, psychophysical or psychological predictors for persistent and recurrent non‐specific LBP. While previous systematic reviews have examined psychological predictors of LBP chronicity (Nieminen et al. 2021) or focused solely on factors such as prior LBP history or demographic characteristics in recurrent LBP (da Silva et al. 2017), non‐have simultaneously investigated both clinical physical, psychophysical or psychological predictors of LBP persistence and recurrence within a single review. The present systematic review and meta‐analysis therefore address this important gap in the literature. In the current review, only studies examining predictors of persistent LBP were identified. Among the psychological predictors examined, depression/distress and fear avoidance demonstrated statistically significant associations with persistent LBP; however, confidence in these findings remains limited due to low certainty of evidence and a high risk of bias across studies. Evidence from a single study suggested that reduced sagittal‐plane flexion and extension velocity and acceleration may be associated with persistent LBP; however, confidence in this finding remains limited, and further replication is required.

The finding that depression‐distress and fear avoidance were significant positive predictors of persistent LBP, aligns with previous systematic reviews and meta‐analyses examining prognostic factors for LBP (Pinheiro et al. 2016; Wertli, Rasmussen‐Barr, et al. 2014; Wong et al. 2022). Moreover, fear avoidance has been reported to be a significant prognostic factor for work‐related outcomes in subacute LBP (Wertli, Eugster, et al. 2014; Wertli, Rasmussen‐Barr, et al. 2014). In contrast, anxiety, catastrophizing, somatisation and passive coping were not significant predictors of persistent LBP, disability or pain intensity. Some individual studies support the role of these psychological constructs in pain persistence. For example, Severeijns et al. (2001) reported that pain catastrophizing significantly predicted pain intensity and disability. Catastrophizing was not identified as a statistically significant predictor in pooled analyses; however, substantial heterogeneity and very low certainty evidence limit definitive conclusion. Additionally, Ramírez‐Maestre et al. (2017) found that anxiety sensitivity was associated with pain catastrophizing, and that catastrophizing had a positive association with disability. However, their cross‐sectional study examined patients with acute LBP, whereas the current systematic review and meta‐analysis synthesized data from longitudinal cohort studies specifically investigating persistent LBP. Somatisation has also been implicated as a factor contributing to the transition from acute to chronic LBP (Pincus et al. 2002). In the current review, passive coping showed only a minimal predictor of persistent LBP. The observed discrepancies may be attributed to differences in outcome measures, LBP duration and population characteristics. Given that anxiety, catastrophizing, somatization and passive coping were not significant predictors of persistent LBP, and considering the high heterogeneity observed across studies, the evidence for these psychological predictors remains inconclusive.

One study identified decreased trunk flexion and extension velocity and acceleration as predictors of persistent LBP. Altered patterns of muscle activation are commonly observed in individuals with LBP, such as increased antagonist muscle activation and decreased agonist muscle activity during movement (D'Hooge et al. 2013) which can directly impact movement velocity and acceleration, consistent with the pain adaptation theory (Lund et al. 1991), which describes systematic changes in muscle recruitment patterns aimed at reducing the amplitude and velocity of potentially painful movements. Based on the limited evidence available, with only two studies examining temporal summation of pain as a predictor of persistent LBP, findings were inconsistent, and no definitive conclusion can be drawn regarding its predictive value. Notably, Overstreet et al. (Overstreet et al. 2021) reported that temporal summation predicted persistent LBP when assessed through movement‐evoked pain, but not for self‐reported pain assessed via questionnaires, suggesting that the method of pain assessment may influence whether temporal summation emerges as a significant predictor. The distinction highlights the importance of standardizing outcome measurement approaches in the future research to better clarify the role of temporal summation in LBP persistence.

To address the potential influence of baseline symptom duration on the identified predictors, findings were examined separately according to whether studies recruited participants with acute or chronic LBP at baseline. When findings were examined according to the chronicity of LBP at baseline, a broadly consistent pattern of psychological predictors was observed across both acute and chronic populations. It should be noted that several included studies recruited mixed populations spanning acute, subacute and chronic LBP and were therefore not included in this narrative chronicity‐based comparison. Among participants who entered studies with acute LBP, fear avoidance, catastrophising and depression were the most consistently identified predictors of persistence (Klyne et al. 2022; Sieben et al. 2005; Swinkels‐Meewisse et al. 2006), with pain self‐efficacy additionally emerging as a protective predictor specific to this group (Klyne et al. 2022). Among participants who already had chronic LBP at baseline, the same psychophysical factors remained relevant predictors (Dunn and Croft 2006; Gomes et al. 2023; Halonen et al. 2019), although the magnitude of prediction appeared larger, particularly for catastrophising (Dunn and Croft 2006), which may reflect the increasing influence of maladaptive cognitive processes as pain becomes more established. The similarity of predictors across acute and chronic LBP populations suggests that fear avoidance, catastrophising and depression may represent important targets for clinical assessment regardless of symptom duration. It should be noted, however, that this comparison was based on a narrative examination of existing reported findings, with only three studies contributing to the acute LBP group and three studies contributing to the chronic group, rather than a formal statistical analysis, and should therefore be interpreted with caution given the heterogeneity in study designs, outcome measures, and follow up periods across included studies. Future research should prospectively examine predictors of persistent LBP separately in acute and chronic populations to determine whether clinically meaningful differences exist.

### 4.1 Methodological Considerations

This systematic review and meta‐analysis has several methodological strengths. This is the first systematic review and meta‐analysis investigating whether physical, psychophysical or psychological factors can predict persistent non‐specific LBP. The protocol was registered in PROSPERO prior to study initiation to ensure transparency and minimize bias. A thorough search strategy was developed with the assistance of a librarian and was implemented with high reviewer agreement. Methodological quality was assessed for all included studies using standardized risk of bias tools and evidence quality assessment frameworks. The analysis combined narrative synthesis with quantitative meta‐analyses when sufficient data were available. Despite these strengths, some limitations must be acknowledged. The included studies demonstrated considerable methodological heterogeneity and population diversity, limiting the feasibility of meta‐analysis to only seven predictors. Furthermore, most of the included studies exhibited high risk of bias, primarily due to attrition, inadequate control of confounding and issues with predictor measurement, which limits confidence in the identified predictors. These findings should therefore be interpreted with caution until confirmed in methodologically rigorous studies. A notable methodological consideration is the heterogeneity of outcome measures across included studies. Pain intensity, disability and composite measures of LBP persistence may not be fully interchangeable as prognostic endpoints and could respond differently to the same predictors. Outcomes were combined due to the limited number of studies available for each individual outcome, and the high heterogeneity observed (*I* ^2^ = 57.1%–95.4%) may partly reflect differences in outcome definitions and measurement approaches. A further consideration is the variability in follow‐up duration across included studies, ranging from 3 months to 6 years. Although all included studies met the minimum follow‐up threshold of 12 weeks, short‐term non‐recovery at 3 months and longer‐term chronicity at 1–6 years may not represent the same clinical construct. Predictors identified at shorter follow‐up periods may reflect early non‐recovery rather than true long‐term persistence, and this variability may partly contribute to the heterogeneity observed across studies. Relatedly, most included studies assessed pain status at discrete follow‐up time‐points rather than continuously, precluding distinction between an isolated flare‐up and pain that had persisted continuously since the initial episode; pain reported at or beyond 12 weeks was therefore assumed to reflect ongoing, unresolved LBP, which may have led to an overestimation of true persistence in some studies. Follow‐up duration should therefore be considered when interpreting individual findings. Related to this, the absence clearly defined and criteria for recurrent LBP represents a further limitation. While some studies used the term ‘recurrent LBP,’ definitions were inconsistent or poorly specified, precluding meaningful evidence synthesis. Upon closer examination, these studies described persistent. Future research should adopt standardized definitions for recurrent LBP to enable comprehensive investigation of its predictors and to improve comparability and clinical applicability. The overall certainty of evidence was predominantly rated as very low to low across most outcomes, further limiting the strength of conclusions that can be drawn. This is compounded by an imbalance in predictor types, with psychological predictors dominated the evidence base, accounting for 12 of 15 included studies, whereas physical predictors were examined in only three studies. Investigations incorporating a wider range of predictor types, including physical measures are needed to advance a more comprehensive understanding of the factors underlying LBP persistence. Finally, considerable variability in baseline LBP status across included studies was observed, with some recruiting participants with acute LBP, others with chronic LBP, and several with unclear baseline pain status. This limits the ability to determine whether the identified predictors apply specifically to patients transitioning from acute to persistent LBP or to those already experiencing chronic LBP. Future research should stratify analyses by baseline LBP status to better clarify the clinical applicability of these findings.

### 4.2 Research and Clinical Implications

Depression‐distress and fear avoidance were supported by low certainty evidence of being predictors of persistent LBP and disability. Unlike fixed demographic or structural factors, these psychological factors are potentially modifiable, supporting the relevance of routinely screening for psychological factors using validated tools such as the Hospital Anxiety and Depression Scale (HADS) and Fear‐Avoidance Beliefs Questionnaire (FABQ) to enable stratified care and timely interventions such as cognitive‐behavioural therapy. However, it is important to acknowledge that a modifiable predictive factor is not necessarily a causal treatment target, and it cannot be assumed that treating these factors will directly prevent LBP persistence. Interventional research is therefore required before these factors can be considered actionable treatment targets in clinical practice. There is currently limited evidence on the value of physical factors in predicting LBP persistence. Future research should prioritize high‐quality prospective studies to examine the relevance physical predictors.

## 5 Conclusion

This systematic review revealed that psychological factors, including depression‐distress and fear avoidance, were significant positive predictors for persistent LBP albeit based on low‐very low certainty of evidence. Regarding physical predictors, only single studies reported that decreased flexion and extension velocity and acceleration may predict persistent LBP, but again, this was based on very low certainty of evidence. Due to the lack of studies investigating physical factors for persistent or recurrent LBP, further research is needed to determine whether physical factors may predict persistent or recurrent LBP, and high‐quality studies are required to enhance the overall certainty of evidence for all predictor domains.

## Author Contributions

All authors contributed to the development of the systematic review and meta‐analysis. K.W., C.W.G.Y. and D.F. served as the first, second and third reviewers, respectively. K.W. and C.W.G.Y. are PhD students, with D.F. as the lead supervisor and M.M. as the co‐supervisor. K.W. constructed the review with guidance from D.F., M.M., C.W.G.Y. and V.D. All authors provided feedback on the preparation of the manuscript for publication. D.F. is the guarantor of the work.

## Funding

K.W. was supported by funding from the Faculty of Medicine, Prince of Songkla University.

## Conflicts of Interest

The authors declare no conflicts of interest.

## Supporting information


**Table S1:** Search strategy by database.

**Table S2:** Quality in Prognosis Studies (QUIPS) form template.

**Table S3:** Reasons for exclusion at full‐text screening.
