# Supplementary material for: Physical, Psychophysical and Psychological Predictors of Persistent or Recurrent Non‐Specific Low Back Pain: A Systematic Review and Meta‐Analysis
Source: Eur J Pain. 2026 Sep 4;30(8):e70358. doi: 10.1002/ejp.70358 (PMC13542868; doi:10.1002/ejp.70358)
Supplement: Supplementary file 1 [file EJP-30-0-s001.pdf]

Table S1. Search strategy by database

1. Search strategy for MEDLINE (OVID interface)	
1. Low back pain	low back pain/
2. Recurrence / Persistence	recurren* or remiss* or repeat* or episod* or relapse
3. Prediction	predict* or prognos* or risk factor* or associat*
4. Cohort studies	longitudinal or cohort or follow up or prospective
5.	1 and 2 and (3 or 4)

2. Search strategy for EMBASE (OVID interface)	
1. Low back pain	low back pain/
2. Recurrence / Persistence	(recurren* or remiss* or repeat* or episod* or relapse).mp
3. Prediction	(predict* or prognos* or risk factor* or associat*).mp
4. Cohort studies	(longitudinal or cohort or follow up or prospective).mp
5.	1 and 2 and 3 and 4

3. Search strategy for APA PsycInfo (OVID interface)	
1. Low back pain	low back pain/
2. Recurrence / Persistence	(recurren* or remiss* or repeat* or episod* or relapse).mp
3. Prediction	(predict* or prognos* or risk factor* or associat*).mp
4. Cohort studies	(longitudinal or cohort or follow up or prospective).mp
5.	1 and 2 and (3 or 4)

4. Search strategy for PubMed	
1. Low back pain	low back pain [MesH]
2. Recurrence / Persistence	recurrence [MeSH] OR recurrent OR remission OR repeat OR episod* OR relapse
3. Prediction	predict* OR prognosis [MeSH] OR risk factor [MeSH] OR associat*
4. Cohort studies	longitudinal OR cohort OR follow up OR prospective
5.	#1 AND #2 AND (#3 OR #4)

5. Search strategy for Cinahl Plus	
1. Low back pain	(MH "Low Back Pain") OR (MH "Back Pain")
2. Recurrence / Persistence	recurren* OR remiss* OR repeat* OR episod* OR relapse
3. Prediction	predict* OR prognos* OR risk factor* OR associat*
4. Cohort studies	longitudinal OR cohort OR follow up OR prospective
5.	S1 AND S2 AND (S3 OR S4)

6. Search strategy for Web of science
(((TS=(low back pain)) AND TS=(recurren* OR remiss* OR repeat* OR episod* OR relapse)) AND TS=(predict* OR prognos* OR risk factor* OR associat*)) AND TS=(longitudinal OR cohort OR follow up OR prospective)

7. Search strategy for Scopus
TITLE-ABS-KEY ("Low Back Pain") AND TITLE-ABS-KEY (recurren* OR remiss* OR repeat* OR episod* OR relapse) AND TITLE-ABS-KEY (predict* OR prognos* OR "risk factor*" OR "associat*") AND TITLE-ABS-KEY (longitudinal OR cohort OR "follow up" OR prospective) AND (LIMIT-TO (DOCTYPE , "ar")) AND (LIMIT-TO (LANGUAGE , "English"))
