# Supplementary material for: Physical, Psychophysical and Psychological Predictors of Persistent or Recurrent Non‐Specific Low Back Pain: A Systematic Review and Meta‐Analysis
Source: Eur J Pain. 2026 Sep 4;30(8):e70358. doi: 10.1002/ejp.70358 (PMC13542868; doi:10.1002/ejp.70358)
Supplement: Supplementary file 2 [file EJP-30-0-s002.pdf]

Table S3. Reasons for Exclusion at Full-Text Screening

**Table Reason 1.** Study designs did not adhere to cohort or longitudinal methodologies (n = 8)	
1	Alshehri MA, van den Hoorn W, Klyne DM, van Dieën JH, Cholewicki J, Hodges PW. Poor lumbar spine coordination in acute low back pain predicts persistent long-term pain and disability. Eur Spine J. 2024;33(6):2380-2394. doi:10.1007/s00586-024-08205-w
2	Chaléat-Valayer E, Denis A, Abelin-Genevois K, et al. Long-term effectiveness of an educational and physical intervention for preventing low-back pain recurrence: a randomized controlled trial. Scand J Work Environ Health. 2016;42(6):510-519. doi:10.5271/sjweh.3597
3	da Silva T, Macaskill P, Kongsted A, Mills K, Maher CG, Hancock MJ. Predicting pain recovery in patients with acute low back pain: Updating and validation of a clinical prediction model. Eur J Pain. 2019;23(2):341-353. doi:10.1002/ejp.1308
4	Denneny D, Walumbe J. Physical activity to prevent recurrences of low back pain. Lancet. 2024;404(10448):98-100. doi:10.1016/S0140-6736(24)01247-9
5	Henschke N, Maher CG, Refshauge KM, et al. Prognosis of acute low back pain: design of a prospective inception cohort study. BMC Musculoskelet Disord. 2006;7:54. Published 2006 Jun 22. doi:10.1186/1471-2474-7-54
6	Kolu P, Suni JH, Tokola K, et al. Neuromuscular exercise and counseling for treating recurrent low back pain in female healthcare workers-Findings from a 24-month follow-up study of a randomized controlled trial. Scand J Med Sci Sports. 2023;33(11):2239-2249. doi:10.1111/sms.14451
7	Leino P, Aro S, Hasan J. Trunk muscle function and low back disorders: a ten-year follow-up study. J Chronic Dis. 1987;40(4):289-296. doi:10.1016/0021-9681(87)90044-0
8	Suni JH, Kolu P, Tokola K, et al. Effectiveness and cost-effectiveness of neuromuscular exercise and back care counseling in female healthcare workers with recurrent non-specific low back pain: a blinded four-arm randomized controlled trial. BMC Public Health. 2018;18(1):1376. Published 2018 Dec 17. doi:10.1186/s12889-018-6293-9

**Table Reason 2.** A mixed population of participants with and without low back pain was included in the same group at recruitment (n = 7)	
1	Adams MA, Mannion AF, Dolan P. Personal risk factors for first-time low back pain. Spine (Phila Pa 1976). 1999;24(23):2497-2505. doi:10.1097/00007632-199912010-00012
2	Claeys K, Dankaerts W, Janssens L, Pijnenburg M, Goossens N, Brumagne S. Young individuals with a more ankle-steered proprioceptive control strategy may develop mild non-specific low back pain. J Electromyogr Kinesiol. 2015;25(2):329-338. doi:10.1016/j.jelekin.2014.10.013
3	Feyer AM, Herbison P, Williamson AM, et al. The role of physical and psychological factors in occupational low back pain: a prospective cohort study. Occup Environ Med. 2000;57(2):116-120. doi:10.1136/oem.57.2.116

4	Gallais L. Low back pain and risk factors for low back pain in car drivers. 2008.
5	Stevenson JM, Weber CL, Smith JT, Dumas GA, Albert WJ. A longitudinal study of the development of low back pain in an industrial population. Spine (Phila Pa 1976). 2001;26(12):1370-1377. doi:10.1097/00007632-200106150-00022
6	Linton SJ, Buer N, Vlaeyen J, Hellsing AL. Are fear-avoidance beliefs related to the inception of an episode of back pain? A prospective study. Psychol Health. 2000;14(6):1051-1059. doi:10.1080/08870440008407366
7	Taanila HP, Suni JH, Pihlajamäki HK, et al. Predictors of low back pain in physically active conscripts with special emphasis on muscular fitness. Spine J. 2012;12(9):737-748. doi:10.1016/j.spinee.2012.01.006

**Table Reason 3.** LBP persistence and recurrent LBP were not measured as outcomes (n = 7)	
1	Burton AK, McClune TD, Clarke RD, Main CJ. Long-term follow-up of patients with low back pain attending for manipulative care: outcomes and predictors. Man Ther. 2004;9(1):30-35. doi:10.1016/s1356-689x(03)00052-3
2	Ferguson SA, Hfes, Hfes H, Ergon S, Human F. Longitudinal observations of low back pain recovery. Chicago, Il, 1998.
3	Koleck M, Mazaux JM, Rascle N, Bruchon-Schweitzer M. Psycho-social factors and coping strategies as predictors of chronic evolution and quality of life in patients with low back pain: a prospective study. Eur J Pain. 2006;10(1):1-11. doi:10.1016/j.ejpain.2005.01.003
4	Lüning Bergsten C, Lundberg M, Lindberg P, Elfving B. Change in kinesiophobia and its relation to activity limitation after multidisciplinary rehabilitation in patients with chronic back pain. Disabil Rehabil. 2012;34(10):852-858. doi:10.3109/09638288.2011.624247
5	Rasmussen-Barr E, Campello M, Arvidsson I, Nilsson-Wikmar L, Ang BO. Factors predicting clinical outcome 12 and 36 months after an exercise intervention for recurrent low-back pain. Disabil Rehabil. 2012;34(2):136-144. doi:10.3109/09638288.2011.591886
6	Ul Hasanat R, Syed SA, Rathore FA, Iftikhar S. Development of a tool for objectively measuring somatic pain in the low back region based on a longitudinal diagnostic study conducted in Karachi, Pakistan. BMJ Open. 2023;13(3):e067129. Published 2023 Mar 16. doi:10.1136/bmjopen-2022-067129
7	Vendrig AA. Prognostic factors and treatment-related changes associated with return to work in the multimodal treatment of chronic back pain. J Behav Med. 1999;22(3):217-232. doi:10.1023/a:1018716406511

**Table Reason 4.** Predictors not related to physical or psychological factors (n = 6)	
1	Cruz EB, Canhão H, Fernandes R, et al. Prognostic indicators for poor outcomes in low back pain patients consulted in primary care. PLoS One. 2020;15(3):e0229265. Published 2020 Mar 27. doi:10.1371/journal.pone.0229265

2	Elders LA, Burdorf A. Prevalence, incidence, and recurrence of low back pain in scaffolders during a 3-year follow-up study. Spine (Phila Pa 1976). 2004;29(6):E101-E106. doi:10.1097/01.brs.0000115125.60331.72
3	Leroux I, Dionne CE, Bourbonnais R. Psychosocial job factors and the one-year evolution of back-related functional limitations. Scand J Work Environ Health. 2004;30(1):47-55. doi:10.5271/sjweh.764
4	Lloyd DC, Troup JD. Recurrent back pain and its prediction. J Soc Occup Med. 1983;33(2):66-74. doi:10.1093/occmed/33.2.66
5	McCracken LM, Gross RT, Sorg PJ, Edmands TA. Prediction of pain in patients with chronic low back pain: effects of inaccurate prediction and pain-related anxiety. Behav Res Ther. 1993;31(7):647-652. doi:10.1016/0005-7967(93)90117-d
6	Stanton TR, Henschke N, Maher CG, Refshauge KM, Latimer J, McAuley JH. After an episode of acute low back pain, recurrence is unpredictable and not as common as previously thought. Spine (Phila Pa 1976). 2008;33(26):2923-2928. doi:10.1097/BRS.0b013e31818a3167

**Table Reason 5.** Conference abstract / poster / oral presentation / appraisal (n = 5)	
1	Chng PPE, Seah HHS, Wong HC. Anxiety and depression predict poorer outcomes in patients with acute low back pain in Singapore. Physiotherapy (United Kingdom) 2015;101: eS244-eS245.
2	Cruz EB, Gomes L, Branco J, Rodrigues AM, Fernandes R, Caeiro C, Paiva S, Pinto I, Moniz R, Canhao H. Low-back pain chronicity in a primary care setting is associated with maladaptive psychosocial factors, other chronic pain condition and high levels of pain at baseline. Annals of the Rheumatic Diseases 2019;78: 2088.
3	Ganesh S. Correspondence: Recurrence of low back pain is common: a prospective inception cohort study. Journal of physiotherapy 2020;66: 64.
4	Lardon A, Dubois JD, Cantin V, Piche M, Descarreaux M. Predictors of long-term disability and absenteeism in a population a worker with low back pain : A longitudinal study. Pain Practice 2016;16: 93.
5	Mitchell T, O'Sullivan P, Burnett A, Straker L, Thornton J, Smith A. Identification of modifiable personal factors that predict low back pain in nursing students: A prospective study. Rheumatology 2009;48: i13.

**Table Reason 6.** Full text not accessible (n = 4)	
1	Biering-Sørensen F. A one-year prospective study of low back trouble in a general population. The prognostic value of low back history and physical measurements. Dan Med Bull. 1984;31(5):362-375.
2	Burton AK and Tillotson KM. Prediction of the clinical course of low-back trouble using multivariable models. Spine 1991;16: 7-14.

3	Deyo RA, Diehl AK. Psychosocial predictors of disability in patients with low back pain. J Rheumatol. 1988;15(10):1557-1564.
4	Rose MJ. The prediction of outcome of an acute episode of low back pain using the fear avoidance model of exaggerated pain perception. 1994.

**Table Reason 7.** Duplicate publication using same cohort; study with smaller sample size excluded to avoid double-counting (n = 2)	
1	Melloh M, Elfering A, Egli Presland C, Roder C, Hendrick P, Darlow B, Theis JC. Predicting the transition from acute to persistent low back pain. Occupational medicine (Oxford, England) 2011;61: 127-131.
2	Melloh M, Elfering A, Stanton TR, Barz T, Aghayev E, Roder C, Theis J-C. Low back pain risk factors associated with persistence, recurrence and delayed presentation. Journal of back and musculoskeletal rehabilitation 2014;27: 281-289.
